# 单操作孔全胸腔镜下肺癌单发肋骨转移切除术

**DOI:** 10.3779/j.issn.1009-3419.2016.04.07

**Published:** 2016-04-20

**Authors:** 诗杰 张, 伟明 黄, 向征 刘, 简 李

**Affiliations:** 100034 北京，北京大学第一医院胸外科 Department of Toracic Surgery, Peking University First Hospital, Beijing 100034, China

**Keywords:** 胸腔镜, 肋骨, 骨肿瘤, 微创手术, Thoracoscopy, Rib, Bone neoplasm, Minimally invasive surgical procedure

## Abstract

**背景与目的:**

随着全胸腔镜手术经验的增加，其适应症也在向复杂的术式延伸。本研究旨在探讨单操作孔全胸腔镜下同期行肺叶联合肋骨切除术的可行性。

**方法:**

结合我科为1例肺癌单发肋骨转移的患者进行了“单操作孔全胸腔镜下肺叶及部分肋骨切除术”进行病例分享并复习有关文献。

**结果:**

肿瘤分期为T1N1M1。患者术后恢复良好并于术后第4天出院。最近一次随访于术后18个月，患者无病生存良好。

**结论:**

筛选后的肺癌伴肋骨转移病例，适合行单操作孔全胸腔镜手术切除。

## Abstract (en)

**Background and objective:**

Complete thoracoscopic surgery has advanced, and its indication has also been extended to complex procedures. The aim of this study is to investigate the feasibility of complete single-utility-port thoracoscopic lobectomy with rib resection.

**Methods:**

A patient was diagnosed with lung cancer and single-rib metastasis. The patient received lobectomy and segment costectomy through complete single-utility-port thoracoscopic surgery. The literature was also reviewed.

**Results:**

The tumor was staged at T1N1M1. The patient made an uneventful recovery and was dismissed on day 4 after surgery. At the last follow-up, the patient was alive and well, with no evidence of the disease at 18 months postoperatively.

**Conclusion:**

Highly selected cases of lung cancer with single-rib metastasis are appropriate candidates for complete single-utility-port thoracoscopic resection.

全胸腔镜手术技术目前在肺癌外科治疗领域已经得到了广泛的应用。随着手术医师经验的增长，一方面胸腔镜手术适应症在逐步扩大，向复杂术士延伸，另一方面为了最大程度地减少患者的创伤和痛苦，操作孔在逐渐减少。单操作孔全胸腔镜手术目前已经有多家中心开展^[1]^，但尚未见涉及骨性胸廓的报告。本研究就一例肺癌单发肋骨转移的患者，在我科成功地接受了单操作孔全胸腔镜下肺叶联合肋骨部分切除手术分享一下体会，并回顾了相关文献。

## 1 材料和方法

### 1.1 材料

患者，女性，53岁，主因“体检发现左肺占位一周”入院。未诉特殊不适，查体无阳性体征。胸部计算机断层扫描（computed tomography, CT）可见左肺下叶直径2 cm分叶状肿物，伴毛刺征，高度怀疑“肺癌”（图 1A）。入院后对患者进行了分期检查。正电子发射型计算机断层显像（positron emission tomography-computed tomography, PETCT）支持“肺癌”的诊断，并且发现左侧第二前肋“溶骨性改变，考虑转移”（图 1B），另外可见左肺门直径8 mm摄取增高的淋巴结。术前心肺功能评估未见异常。

**1:**
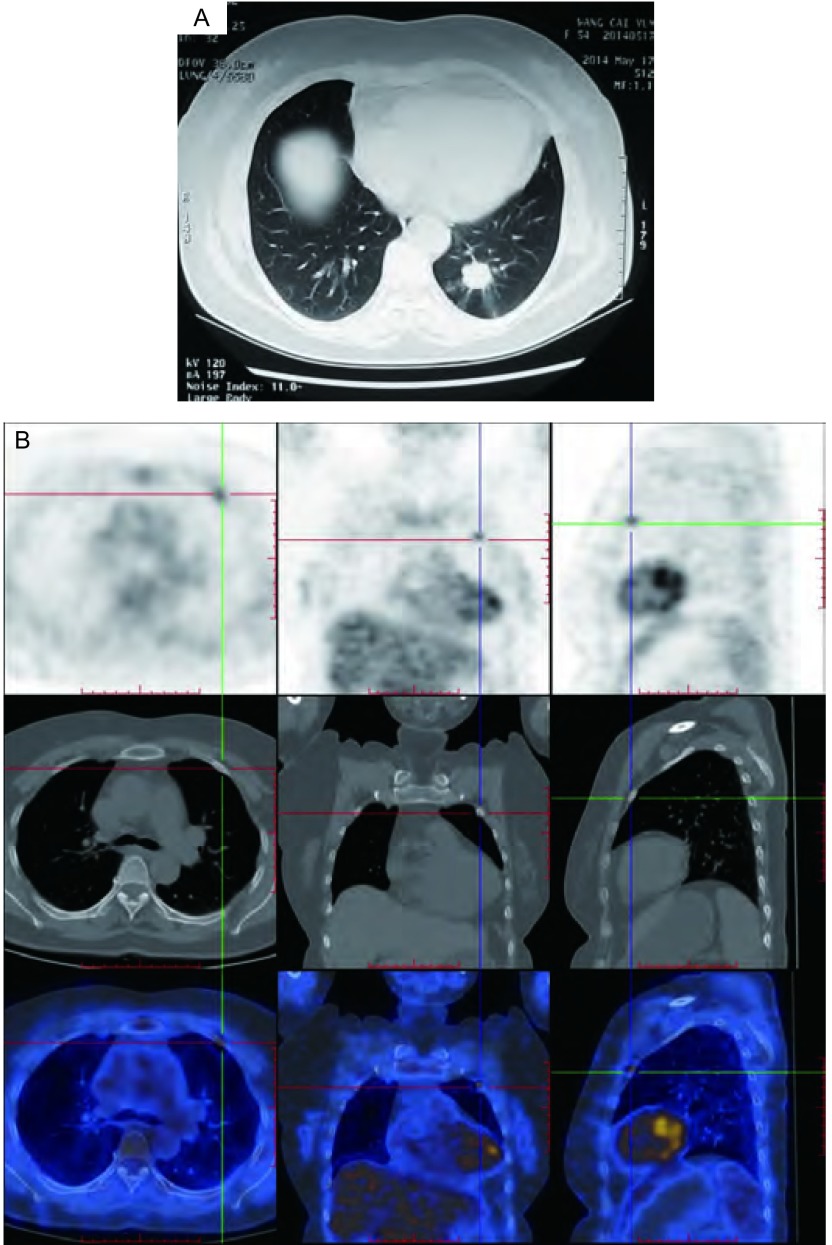
患者临床影像学特征。A：胸部CT显示左肺下叶肿瘤。B：PET-CT示左侧第二肋骨溶骨性病变伴代谢增高。 Clinical radiologic features of the patient. A: Chest CT scan image demonstrating the tumor located in left lower lobe. B: PET-CT scan demonstrating osteolytic lesion in the left 2^nd^ rib with heterogeneous radiotracer uptake. CT: computed tomography; PET-CT: positron emission tomography-computed tomography.

患者TNM分期为cT1N1M1，属Ⅳ期肺癌，一般来讲不具有手术指征。但该患者一般状况好，肋骨转移为单发，且与原发灶在同一侧，根据2014版《肺癌骨转移诊疗专家共识》的指导意见^[2]^，我们认为该患者仍然可以接受手术治疗。

### 1.2 方法

该患者的手术方案为“左肺下叶切除、纵隔淋巴结清扫及左第二肋骨部分切除术”。为减少患者的创伤和痛苦，我们采用单操作孔全胸腔镜手术。手术在全麻双腔气管插管下进行，右侧卧位，左第7肋间腋后线做1.5 cm观察孔，置入胸腔镜。左腋前线第4肋间做3 cm操作口，放入切口保护套。

手术分两部分进行，第一部分，先按肺静脉-叶间裂-肺动脉-支气管的方式切除左肺下叶，冰冻病理明确为“腺癌”，再清扫肺门及同侧纵隔淋巴结。第二部分，转移的第二肋骨部分切除。在胸腔镜引导下定位第二肋骨，可见病灶处肋骨欠平整，范围约1 cm，并以手指触诊进一步确认。以电刀分离肋骨上下缘的肋间组织（图 2A），此处肋间血管较细，用电刀直接凝断。以长弯钳分离肋骨下方，只充分分离拟切断处即可。于肋骨下方用长弯钳导入钢丝锯（图 2B），用Kocher钳固定线锯两端。由于操作空间有限，Kocher钳固定点距肋骨2 cm-3 cm左右比较合适。尽量垂直肋骨稍用力拉动钢丝锯（图 2C），肋骨即可离断。患者第二前肋切除长度约为8 cm（图 2D），胸骨端断点在肋软骨处。创面充分止血后，冲洗胸腔，试水无漏气，留置胸腔引流管1根后关胸。

**2:**
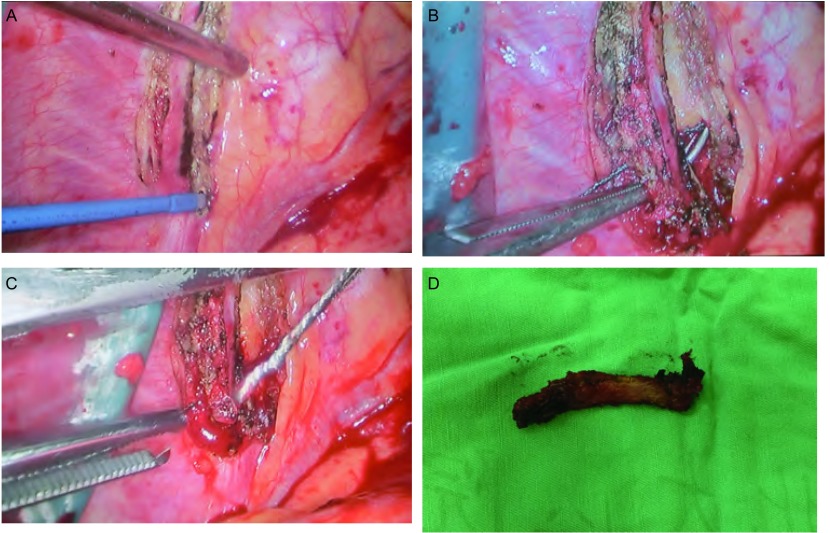
单操作孔全胸腔镜下肋骨部分切除的术中照片。A：用电刀分离肋间组织。B：长弯钳导入钢丝锯。C：由Kocher钳固定钢丝锯并锯断肋骨。D：切除的部分肋骨。 Intraoperative pictures of single utility port completely thoracoscopic segment costec tomy. A: The intercostal tissueis dividing with the electrocautery. B: The Gigli saw is positioning at the target area with the curved clamp. C: The rib is severing with a Gigli saw held by Kocher clamps. D: The resected rib segment.

## 2 结果

患者手术过程顺利，术后第4天拔除胸腔引流管出院，总引流量约为470 mL，术后恢复顺利，无并发症。术后石蜡病理证实肺浸润性腺癌，T1N1M1，并行表皮生长因子受体（epidermal growth factor receptor, *EGFR*）基因检测，于21位点检测到突变。术后2周开始口服吉非替尼。最后一次随访为术后18个月，患者生存情况良好，体力状况评估采用美国东部肿瘤协作组（Eastern Cooperative Oncology Group, ECOG）活动状态评分表，评分为1分，并未发现肿瘤复发及转移征象。

## 3 讨论

胸腔镜手术一直是胸外科的热点话题，国内全胸腔镜下操作在胸外科手术治疗中的比重在逐渐增加，开展的单位也越来越多。手术方式也从最初的简单肺叶切除到现在很多单位开展的肺叶袖式切除等^[3]^。但胸腔镜手术治疗肋骨的病变，国内罕见文献报道。国内一篇涉及真正胸腔镜肋骨切除的文章是经两个操作孔，只涉及原发肋骨肿瘤，且都是后肋^[4]^。而且以往大家认为适合的病例并不多，但现在陆续有文献报道对于胸壁受侵犯的肺癌整块切除，肋骨的转移瘤也可成为胸腔镜手术的适应症^[5-8]^。

随着对减少患者创伤和痛苦的要求，腔镜技术的操作孔也在逐渐减少，单操作孔全胸腔镜手术近年来已在很多单位开展，在我科也是一项成熟技术。对于本例患者，由于受累的肋骨正好位于原发灶同一侧，且为单根肋骨单处病变，情况相对简单，所以我们尝试了单操作孔全胸腔镜下操作。Gonzalez^[5]^曾报道过一例单操作孔切除侵犯胸壁的周围型肺癌病例，但其属于杂交方法，即后孔切除胸壁，前孔切除肺叶。而我们在实践中发现完全可以在一个操作孔内完成肺叶和部分肋骨切除手术。

国内目前还没有单位开展“单操作孔全胸腔镜下肋骨切除术”的报道。其难点主要是器械的不匹配，国外有专门的内镜用肋骨剪或肋骨钻^[6, 7]^，而国内很少有单位去购买这些目前不常用到的器械，此时，钢丝锯起到了很关键的作用。钢丝锯（Gigli saw）为神外及骨科常用器械，胸外科不常用到。在Demmy等^[8]^总结的腔镜切除胸壁及重建的策略中有所提及。内镜下游离肋骨并不困难，此时注意不要将拟切除肋骨段全部游离，这样中间的组织对于肋骨起到固定作用，方便离断。在预定肋骨断点下方穿过钢丝锯，Kocher钳固定两端，由于第二肋距第四肋的操作孔较近，所以普通的Kocher钳可以应用。操作时尽量垂直肋骨适当用力反复拉动钢丝锯，幅度不要过大，以免造成内脏的副损伤，需要一定的耐心，肋骨可以很快离断。经胸内进行肋骨切除的优势在于除了表面没有切口，更重要的是对于胸壁肌肉软组织的影响降到最低，有利于呼吸功能的恢复。而且在胸腔镜的辅助下，钢丝锯具有良好的导向性，肋骨切除范围几乎没有死角，但是我们的经验更易于切除前肋及侧肋。对于后肋累及或接近肋骨小头的病灶就不适合钢丝锯切除^[7]^，因为需要切除部分胸椎结构。另外，对于胸壁的病变，胸腔镜切除的重要性还在于判断有无胸膜的播散以及从胸膜面决定切除范围。一般而言，肋骨转移性病变的切缘要求在2 cm以上^[9]^。

目前在胸腔镜手术的适应症中，尽管肺癌涉及骨性胸壁的病变并未提及。但是从文献回顾和我们的经验看都是可行的。首先从治疗的生存获益来看，恶性肿瘤的单发肋骨转移国内外都有学者建议采取手术切除^[2, 10, 11]^。对于肺癌肋骨转移手术后也有长期存活的报道^[11]^，而且本例患者目前随访18个月，仍然无病生存良好。其次从胸腔镜切除肋骨技术的可行性看，通过钢丝锯的使用，是可以比较容易地经单操作孔全胸腔镜手术完成。因此我们认为经过严格的病例筛选，肺癌伴单发肋骨转移病例，是适合行单操作孔全胸腔镜手术切除的。
